# Anticatabolic and Anti-Inflammatory Effects of Myricetin 3-O-β-d-Galactopyranoside in UVA-Irradiated Dermal Cells via Repression of MAPK/AP-1 and Activation of TGFβ/Smad

**DOI:** 10.3390/molecules25061331

**Published:** 2020-03-14

**Authors:** Jung Hwan Oh, Fatih Karadeniz, Jung Im Lee, So Young Park, Youngwan Seo, Chang-Suk Kong

**Affiliations:** 1 Marine Biotechnology Center for Pharmaceuticals and Foods, College of Medical and Life Sciences, Silla University, Busan 46958, Korea; wjdghks0171@naver.com (J.H.O.); karadenizf@outlook.com (F.K.); think3433@daum.net (J.I.L.); 2 Department of Food and Nutrition, College of Medical and Life Sciences, Silla University, Busan 46958, Korea; qkrthdud0409@naver.com; 3 Division of Marine Bioscience, College of Ocean Science and Technology, Korea Maritime and Ocean University, Busan 49112, Korea; ywseo@kmou.ac.kr; 4 Department of Convergence Study on the Ocean Science and Technology, Ocean Science and Technology School, Korea Maritime and Ocean University, Busan 49112, Korea

**Keywords:** HaCaT keratinocyte, HDF, MMP-1, photoaging, UVA

## Abstract

UV irradiation is one of the main causes of extrinsic skin aging. UV-mediated skin aging, also known as photoaging, causes excessive breakdown of extracellular matrix which leads skin to lose its elasticity and strength. Several phytochemicals are known to exert anti-photoaging effects via different mechanisms, partly due to their antioxidant properties. The current study has been carried out to determine the potential anti-photoaging properties of myricetin 3-O-β-d-galacto-pyranoside (M3G), a flavonol glycoside isolated from *L. tetragonum*, in UVA-irradiated in vitro models; HaCaT keratinocytes and human dermal fibroblasts (HDFs). UVA-induced changes in MMP-1 and collagen production have been observed in HaCaT keratinocytes and HDFs. Further, UVA-induced activation of MAPK signaling, and pro-inflammatory cytokine production have been investigated. TGFβ/Smad pathway has also been analyzed in UVA-irradiated HDFs. Treatment with M3G reversed the UVA-induced changes in MMP-1 and collagen production both in HaCaT keratinocytes and HDFs. UVA-mediated activation of p38, ERK and JNK MAPK activation was also inhibited by M3G treatment in HaCaT keratinocytes. In HDFs, M3G was able to upregulate the TGFβ/Smad pathway activation. In addition, M3G downregulated the UVA-induced pro-inflammatory cytokines in keratinocytes and HDFs. It has been suggested that the M3G has exerted potential antiphotoaging properties in vitro, by attenuating UVA-induced changes in MMP-1 and collagen production in keratinocytes and dermal fibroblasts.

## 1. Introduction

Photoaging is the extrinsic aging of the human skin caused by ultraviolet radiation. Photoaging manifests itself with wrinkles and loss of elasticity due to increasing collagen and elastin damage and inflammatory response in the skin [1]. UV radiation has three subtypes categorized as UVA, UVB and UVC. Ozone layer almost entirely hinders arrival of UVC to be a factor in photoaging whereas UVA and UVB can reach and penetrate human skin [2,3]. Although solar UV irradiation is needed for the formation of vitamin D in humans, chronic exposure damages skin. UVA has the ability to penetrate the skin layer deeper than UVB [2]. This penetration leads to cellular damage in both epidermis and dermis layer as opposed to UVB which mainly affects epidermis and upper part of the dermis. UVA irradiation is documented to be one the main causes of premature skin aging, formation of wrinkles and some types of skin cancer. UVA exposure leads to detrimental changes in cellular pathways of the dermal fibroblasts where collagen and elastin degrading enzymes, matrix metalloproteinases (MMPs) and inflammatory response are stimulated via activator protein 1 (AP-1) mediated signaling [4,5]. As it penetrates deep parts of dermis, UVA exposure causes damages in dermal fibroblasts, where the collagen is produced to give skin its firmness [6]. Increasing production of MMPs and diminished collagen formation are among main reasons behind UVA mediated damage in dermal layer.

Cellular response to UVA irradiation is evidently governed by the excessive oxidative stress due to production of the reactive oxygen species (ROS) as well as reactive nitrogen species (RNS) mainly nitric oxide (NO) [7,8]. It has been shown that the production of RNS contributes greatly to the UVA-induced DNA damage, especially inflammatory response and MMP production and therewith damages to extracellular matrix (ECM) stability [8]. In addition, UVA-induced increase in ROS and RNS triggers a set of signaling which consists of the activation of mitogen-activated protein kinase (MAPK) cascade responsible for the MMP mediated deterioration in ECM stability. Activation of p38 kinase, c-Jun *N*-terminal kinase (JNK) and extracellular signal-regulated kinase (ERK) MAPKs regulates the UVA-induced transcriptional changes. Concomitant activation of these MAPKs regulates the transcriptional activity of the transcription factor AP-1 which is a heterodimer of c-Jun and c-Fos proteins [9,10]. UVA mediated changes in MMP and collagen productions are regulated by the AP-1 activity which depends on the phosphorylation and subsequent nuclear translocation of c-Jun and c-Fos.

**Figure 1:**
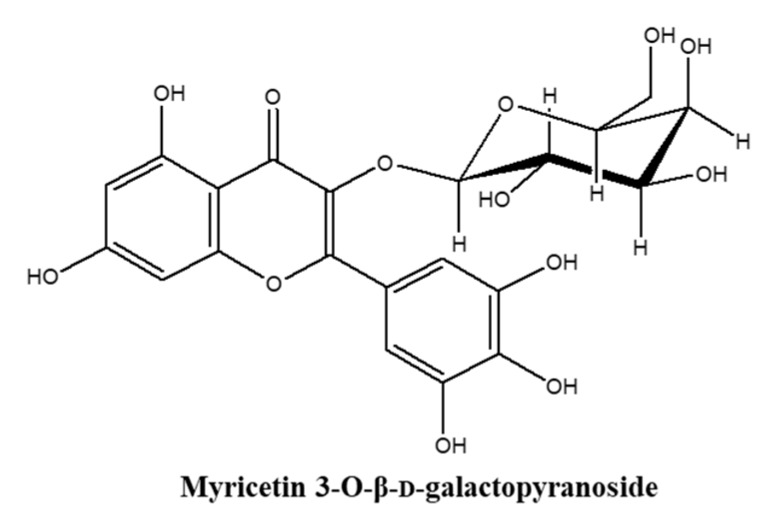
Chemical structure of myricetin 3-O-β-d-galactopyranoside (M3G).

Flavonoids are phytochemicals with various documented biologically active properties and found as flavonoid glycosides in their natural forms. Up to date about 6000 flavonoids have been identified and several studies have reported miscellaneous bioactivities in addition to their roles in plants [11,12]. Many medicinal, nutritional and cosmetic products contain different types of flavonoid derivatives due to their promising beneficial effects on health such as anti-inflammatory, antioxidant, antibacterial, anti-carcinogenic and antiviral properties [12]. Studies have described numerous flavonoids with notable cosmeceutical potential with different abilities such as skin whitening, anti-wrinkle, UV absorption, anti-inflammation and skin moisturizing [13]. Different types of flavonoid derivatives are being isolated and screened for their bioactivities constantly. Halophytes are salt marsh plants that endure high salinity conditions; therefore, produce high amounts of antioxidant phytochemicals including flavonoid derivatives. *Limonium tetragonum* is an edible halophyte growing in salt marshes and rocky shores of Korea and known to possess health beneficial properties including but not limited to antioxidative, hepatoprotective and antitumor activities [14,15]. As a part of ongoing research on bioactive flavonoids, myricetin 3-O-β-d-galactopyranoside (M3G) (Figure 1) has been isolated from *L. tetragonum* and its MMP inhibitory [16], anti-melanogenic [17] and anti-adipogenic [18] properties have been reported. However, its effects against UVA-mediated damages in skin cells remain unknown to the best of our knowledge. Thus, present study has been carried out to investigate the effects of M3G on the UVA-mediated production of inflammatory cytokines, MMPs and type I procollagen and their underlying mechanism in HaCaT keratinocytes and human dermal fibroblast (HDFs).

## 2. Results

### 2.1. Effect of UVA Irradiation and M3G on the Viability of HaCaT Keratinocytes and HDFs

**Figure 2:**
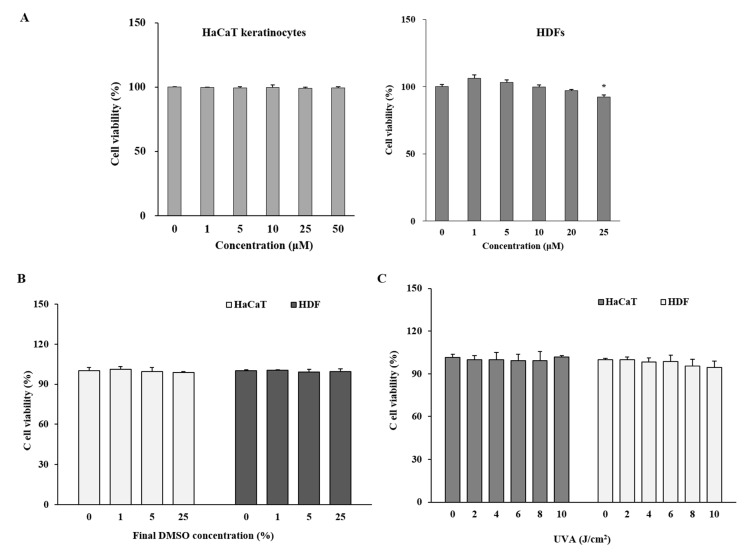
Effect of myricetin 3-O-β-d-galactopyranoside (M3G), vehicle (10% DMSO) and UVA on the viability of HaCaT keratinocytes and human dermal fibroblasts (HDFs). HaCaT keratinocytes and HDFs were treated with given final concentrations of M3G (**A**) and equivalent amount of DMSO (**B**) and incubated for 24 h. Viability of the cells were then quantified by MTT assay. (**C**) HaCaT keratinocytes and HDFs were irradiated with UVA (0–10 J/cm^2^) and incubated for 24 h and the viability of the cells were quantified by MTT assay. Relative cell viability was expressed as the mean ± SD (*n* = 3) (% of untreated control) of three independent experiments run in triplicate. * *p* < 0.05 compared to untreated control group.

Any potential cytotoxicity of UVA irradiation and M3G on HaCaT keratinocytes and HDFs was evaluated by MTT assay. Both cells were incubated with M3G for 24 h without UVA irradiation. Cells were also irradiated by UVA and incubated for 24 h without M3G treatment to assess the cytotoxic effects of UVA irradiation. In addition, vehicle only treatment was carried out using the same volume of 10% dimethyl sulfoxide (DMSO, in distilled water) vehicle. Effects on cell viability were analyzed by comparison with untreated control group. Treatment with M3G did not show any cytotoxicity in HaCaT keratinocytes for the doses up to 25 μM (Figure 2A). However, HDFs exhibited a 7.57% decrease in cell viability following 25 μM M3G treatment. Both cells did not show any drop in cell viability following vehicle treatment with final concentrations of 0.01%, 0.05% and 0.25% DMSO which was equivalent amount of M3G final concentrations of 1, 5 and 25 μM, respectively in wells (Figure 2B). Hantke et al. [19] reported that these concentrations did not affect the UVA-induced changes of MMP expression in both HaCaT keratinocytes and HDFs. In addition, the highest final concentration of DMSO (0.25%) as a vehicle were comparable to reported studies where similar concentrations of DMSO did not exert any effects in cultured cells against UVA-induced changes [20,21]. UVA doses up to 10 j/cm^2^ did not show any cytotoxicity in both HaCaT and HDFs after 24 h incubation (Figure 2C). According to results, further assays used the M3G concentration of 25 μM and below, given that the concentrations higher than 25 μM in HDFs might lower the cell viability below 90% compared to untreated control.

### 2.2. Effect of M3G on the Release of MMP-1 and Type Iα1 Procollagen in UVA-Irradiated HaCaT Keratinocytes and HDFs

**Figure 3:**
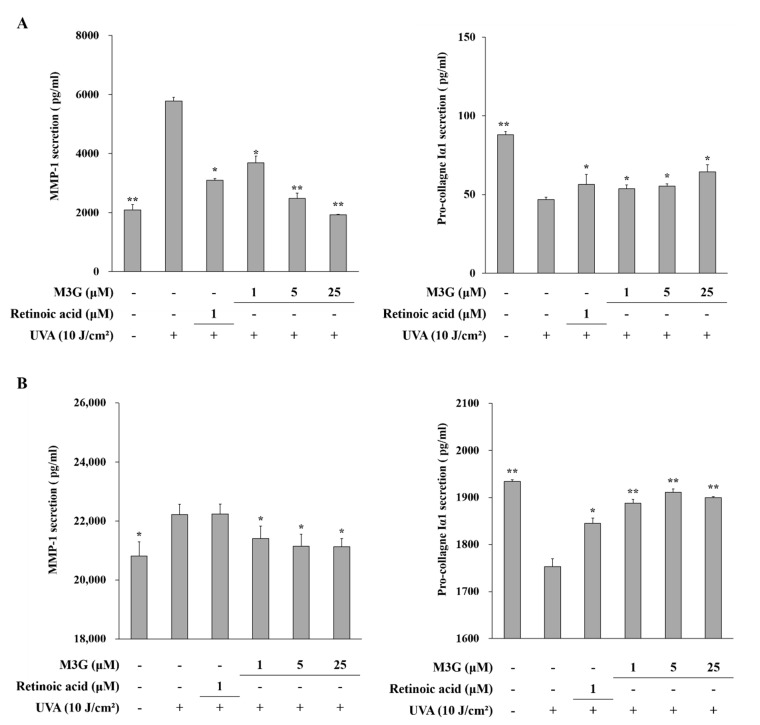
Effect of M3G on the UVA-induced release of MMP-1 and procollagen Iα1 in HaCaT keratinocytes (**A**) and HDFs (**B**). Cells were exposed to UVA radiation (10 J/cm^2^) and treated with given concentrations of M3G. After 24 h incubation, MMP-1 and pro-collagen Iα1 release in the cell culture media was quantified by ELISA. Retinoic acid was used as a positive control. Values are means ± SD of three independent experiment run in triplicate. * *p* < 0.05, ** *p* < 0.01 compared to UVA-irradiated untreated control.

The effect of M3G on the UVA-mediated changes of MMP-1 and type Iα1 procollagen production was determined using an ELISA to quantify the secretion levels. UVA irradiation (10 J/cm^2^) resulted in increased production of MMP-1 and decreased production of type Iα1 procollagen in both HaCaT keratinocytes and HDFs (Figure 3A,B). MMP-1 release was increased from 2090.0 pg/mL to 5782.7 pg/mL in HaCaT keratinocytes and from 20822.7 pg/mL to 22228.0 pg/mL in HDFs following UVA irradiation. Treatment with M3G inhibited the release of MMP-1 in UVA irradiated HaCaT keratinocytes by 66.6% (1930.7 pg/mL) at the concentration of 25 μM (Figure 3A). In HDFs, M3G (25 μM) decreased the MMP-1 release by 4.8% (21143.6 pg/mL) (Figure 3B). Pro-collagen Iα1 release was decreased from 88.0 pg/mL to 46.9 pg/mL in HaCaT keratinocytes and from 1933.9 pg/mL to 1753.3 pg/mL in HDFs following UVA irradiation. M3G (25μM) increased the type I procollagen production in UVA-irradiated HaCaT keratinocytes by 37.2% (64.4 pg/mL) (Figure 3A) and in UVA-irradiated HDFs by 9.0% (1911.5 pg/mL) (Figure 3B).

### 2.3. Effect of M3G on the Production of Nitric Oxide (NO) in UVA-Irradiated HaCaT Keratinocytes

**Figure 4:**
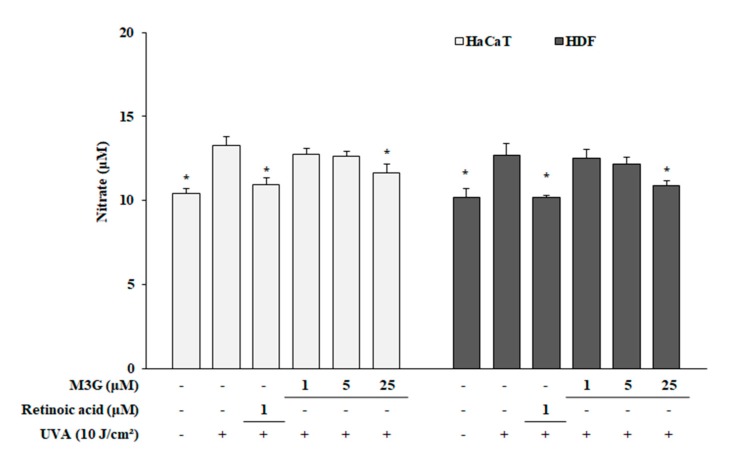
Effect of M3G on the UVA-induced generation of nitric oxide in HaCaT keratinocytes and HDFs. Cells were exposed to UVA radiation (10 J/cm^2^) and treated with given concentrations of M3G. After 24 h incubation, nitric oxide generation was analyzed as the nitrate amount in the cell culture medium. Retinoic acid was used as a positive control. Values are means ± SD of three independent experiment run in triplicate. * *p* < 0.05 compared to UVA-irradiated untreated control.

In order to investigate any possible effect of M3G on the UVA-induced reactive species production in HaCaT keratinocytes and HDFs, nitrite oxide was measured in cultured cell media as amount of nitrate. Results have showed that UVA irradiation increased the NO production in both HaCaT keratinocytes and HDFs. Following UVA irradiation, nitrate amount was increased from 10.43 to 13.27 in HaCaT keratinocytes and from 10.21 to 12.71 in HDFs. Treatment with M3G (25 μM) decreased nitrate concentration to 11.63 in HaCaT keratinocytes and to 10.92 in HDFs (Figure 4).

### 2.4. Effect of M3G on the Expression of MMP-1, MMP-9 and Type I Procollagen in UVA-Irradiated HaCaT Keratinocytes

**Figure 5:**
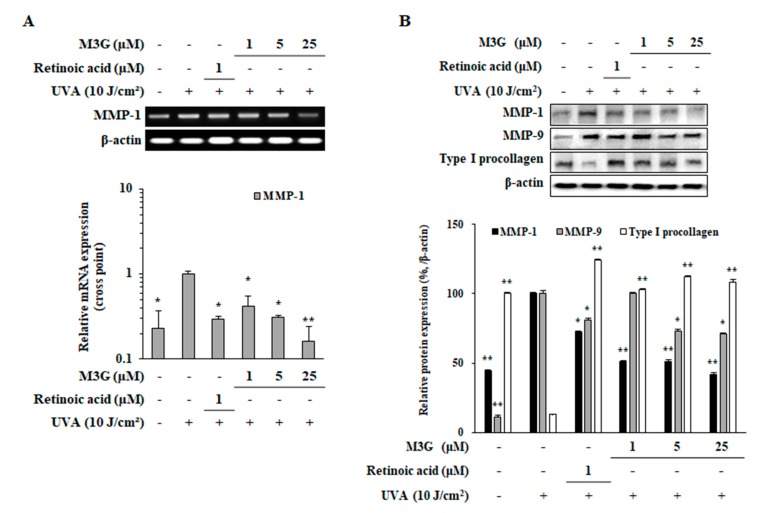
Effect of M3G on the expression of MMP-1, -9 and type I procollagen in UVA-irradiated HaCaT keratinocytes. Cells were exposed to UVA radiation (10 J/cm^2^) and treated with given concentrations of M3G. After 24 h incubation, the mRNA expression of MMP-1 was analyzed with RT-PCR (upper panel) and RT-qPCR (lower panel) (**A**). RT-qPCR expression quantity was given relative to the untreated UVA-irradiated control, calculated by 2^−(ΔΔCt)^ method. Protein levels of MMP-1, -9 and type I procollagen were determined by Western blotting (**B**). β-Actin was used as an internal control for RT-PCR, RT-qPCR and Western blotting. Retinoic acid was used as a positive control. Values are means ± SD of three independent experiment run in triplicate. * *p* < 0.05, ** *p* < 0.01 compared to UVA-irradiated untreated control.

To determine the effect of M3G on the mRNA expression of MMP-1 and protein expression of MMP-1, MMP-9 and type I procollagen in UVA-irradiated (10 J/cm^2^) HaCaT keratinocytes, RT-PCR, RT-qPCR and Western blotting were utilized. Presence of M3G (1, 5, 25 μM) in UVA-irradiated HaCaT keratinocytes dose-dependently inhibited the mRNA expression of MMP-1 analyzed by both RT-PCR and RT-qPCR (Figure 5A). MMP-1 expression was increased 4.35-fold by UVA irradiation which was decreased by M3G treatment (25 μM) by 83.8%. Protein expression levels were observed to be parallel with mRNA expression where M3G treatment decreased the UVA-related increase in MMP-1 levels by 58% (Figure 5B). In addition, protein levels of MMP-9 and type I procollagen were also investigated. UVA irradiation caused upregulation (89.7%) in MMP-9 protein expression and suppression (87.9%) in type I procollagen levels both of which were reverted by M3G treatment by 29.8% and 95.0%, respectively.

### 2.5. Effect of M3G on the UVA-Induced Production of Pro-Inflammatory Mediators in HaCaT Keratinocytes

**Figure 6:**
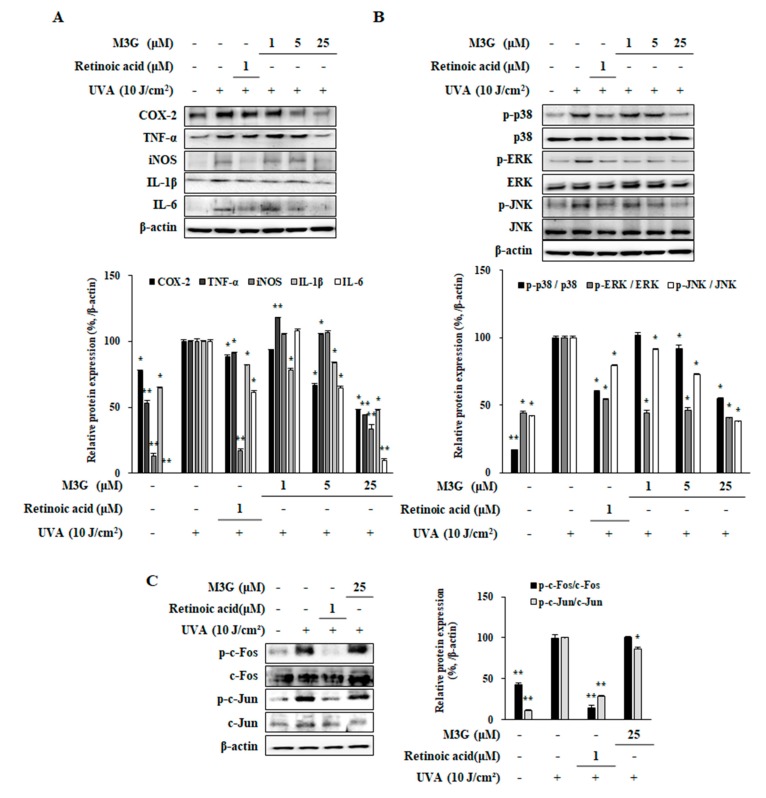
Effect of M3G on the production levels of pro-inflammatory mediators (**A**) and on the phosphorylation of MAPK/AP-1 signaling (**B**, **C**) in UVA-irradiated HaCaT keratinocytes. Cells were exposed to UVA radiation (10 J/cm^2^) and treated with given concentrations of M3G. After 24 h incubation, the protein levels of; pro-inflammatory mediators (COX-2, TNF-α, iNOS, IL-1β and IL-6) (**A**), phosphorylated (p-) and inactive forms of p38, ERK and JNK MAPKs (**B**), and p- and inactive form of c-Fos and c-Jun (**C**) proteins were determined by Western blotting. Values are means ± SD of three independent experiments. * *p* < 0.05, ** *p* < 0.01 compared to UVA-irradiated untreated control.

Effect of M3G on the UVA-mediated inflammatory response in HaCaT keratinocytes was analyzed by investigating the protein expression levels of pro-inflammatory enzymes and cytokines (cyclooxygenase-2 (COX-2), inducible nitric oxide synthase (iNOS), tumor necrosis factor α (TNF-α), interleukin-1β (IL-1β) and IL6) utilizing Western blotting. Results indicated that UVA exposure (10 J/cm^2^) caused a notable increase in pro-inflammatory response in HaCaT keratinocytes (COX-2, 22.6%; TNF-α, 46.6%; iNOS, 86.8%; IL-1β, 35.5%; IL-6, 98.9%). M3G treatment decreased the levels of COX-2, iNOS, TNF-α., IL-1β and IL6 levels by 51.7%, 55.9%, 66.6%, 52.6% and 81.3%, respectively, at the concentration of 25 μM (Figure 6A). Results suggested that M3G was able to suppress the UVA-induced inflammation in keratinocytes via inhibiting the production of inflammatory cytokines.

### 2.6. Effect of M3G on the Phosphorylation of MAPKs, c-Fos and c-Jun in UVA-Irradiated HaCaT Keratinocytes

Effect of M3G on the UVA-stimulated activation of MAPK signaling was investigated through phosphorylation levels of AP-1 regulating MAPKs using Western blotting. UVA irradiation (10 J/cm^2^) resulted in increased activation of MAPKs, shown as significantly elevated phosphorylation of p38, ERK and JNK MAPKs (Figure 6B). Following UVA irradiation, relative phosphorylation of MAPKs (normalized against their total protein levels) was increased by 82.8% for p38, 56.1% for ERK and 58.0% for JNK compared to non-irradiated group. Levels of phosphorylation were significantly lowered by 45.1% for p38, 59.2% for ERK and 62.0% for JNK in M3G treated (25 μM) HaCaT keratinocytes. Next, phosphorylation levels of c-Fos and c-Jun, building blocks of AP-1 transcription factor, were analyzed in UVA irradiated HaCaT keratinocytes (Figure 6C). UVA exposure increased the phosphorylation of c-Fos and c-Jun by 51.3% and 88.6%, respectively, compared to non-irradiated control. M3G treatment at 25 μM concentration significantly decreased the phosphorylation level of c-Jun by 13.2% but failed to affect the c-Fos phosphorylation. Results demonstrated that M3G suppressed MAPK mediated activation of AP-1 (via c-Jun) suggesting that this led to inhibition of MMP-1 production.

### 2.7. Effect of M3G on the UVA-Induced Expression of MMPs and Type I Procollagen in HDFs

**Figure 7:**
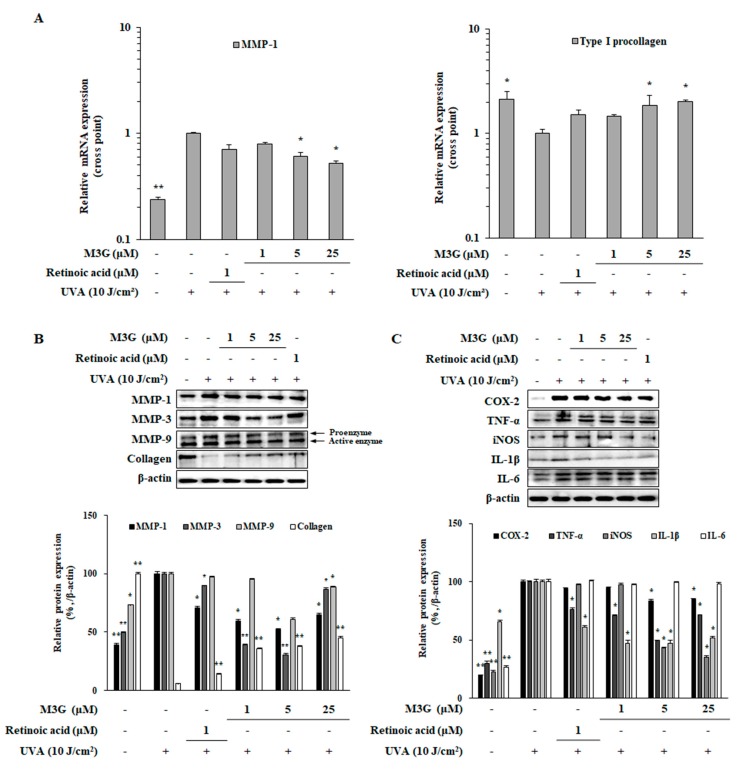
Effect of M3G on the expression of MMPs, type I procollagen and pro-inflammatory cytokines in UVA-irradiated HDFs. Cells were exposed to UVA radiation (10 J/cm^2^) and treated with given concentrations of M3G. After 24 h incubation cells were harvested for RT-qPCR and Western blotting. The mRNA expressions of MMP-1 and type I procollagen were determined by RT-qPCR and given as the relative expression quantity compared to untreated UVA-irradiated group (**A**). Protein levels of; MMP-1, -3, -9 and collagen (**B**) as well as pro-inflammatory mediators (COX-2, TNF-α, iNOS, IL-1β and IL-6) (**C**) were investigated by Western blotting. Values are means ± SD of three independent experiment run in triplicate. * *p* < 0.05, ** *p* < 0.01 compared to UVA-irradiated untreated control.

To investigate the effects of M3G on the mRNA and protein expressions of MMPs and collagen in UVA-irradiated dermal fibroblasts, RT-qPCR and Western blotting were utilized, respectively. UVA-irradiated HDFs expressed 4.21-fold higher MMP-1 mRNA compared to non-irradiated control group whereas type I procollagen mRNA was expressed 2.13-fold less (Figure 7A). At the concentration of 25 μM, M3G treatment suppressed MMP-1 mRNA expression by 1.90-fold and upregulated type I procollagen mRNA expression by 2.03-fold. UVA-induced increase in protein levels of MMP-1 (60.7%) and decrease in collagen (94.2%) were also ameliorated in a similar manner by M3G treatment (Figure 7B). At 25 μM, M3G treatment decreased MMP-1 protein levels by 47.5% and increased collagen levels by 39.2%. In addition, UVA-induced increase (50.4%) in MMP-3 levels were slightly decreased by 25 μM M3G treatment. However, M3G was only able to decrease the levels of MMP-9 proenzyme not the active enzyme suggesting that the effect of M3G on the suppression of MMPs is not specific to MMP-1 and also follows a common transcriptional activation of MMP-1, -3 and -9 rather than enzyme specific inhibition.

### 2.8. Effect of M3G on the UVA-Induced Inflammatory Response in HDFs

To determine the effects of M3G on the inflammation in dermal fibroblasts caused by UV exposure, HDFs were irradiated by UVA (10 J/cm^2^) and the protein levels of pro-inflammatory enzymes and cytokines (COX-2, iNOS, TNF-α, IL-1β and IL6) were investigated by Western blotting. Similar to its effects in HaCaT keratinocytes, UVA exposure (10 J/cm^2^) significantly increased the protein levels of COX-2, iNOS, TNF-α, IL-1β and IL6 which were decreased by M3G treatment, except IL6 (Figure 7C). M3G (25 μM) treated HDFs expressed 16.3%, 50.5%, 57.2%, 52.8% and 0.8% less amounts of COX-2, iNOS, TNF-α, IL-1β and IL6 proteins, respectively compared to UVA-irradiated only cells. Results suggested that M3G hindered the inflammatory response in UVA-irradiated HDFs via suppression of inflammatory cytokine production.

### 2.9. Effect of M3G on the Activation of MAPKs and AP-1 in UVA-Irradiated HDFs

**Figure 8:**
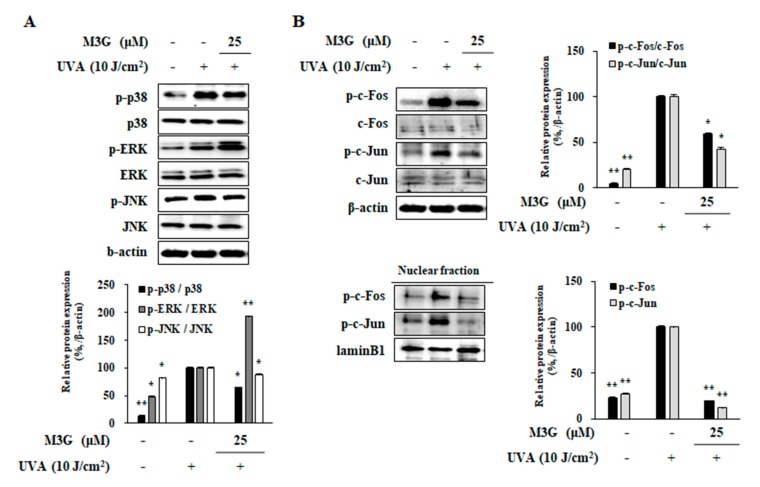
Effect of M3G on the phosphorylation of MAPK and AP-1 in UVA-irradiated HDFs. Cells were exposed to UVA radiation (10 J/cm^2^) and treated with 25 μM of M3G. After 24 h incubation cells were harvested and the MAPK signaling (phosphorylated (p-) and inactive forms of p38, ERK and JNK MAPKs) (**A**) and AP-1 activation (p- and inactive forms of c-Fos and c-Jun in whole cell and nuclear fractions) (**B**) were investigated by Western blotting. Values are means ± SD of three independent experiments. * *p* < 0.05, ** *p* < 0.01 compared to UVA-irradiated untreated control.

As a suggested mechanism for the effects of M3G against UVA induced changes in HaCaT keratinocytes, UVA mediated activation of MAPKs was also investigated in HDFs. Treatment with M3G (25 μM) inhibited the UVA-induced (10 J/cm^2^) increase of p38 and JNK phosphorylation by 35.3% and 12.9%, respectively (Figure 8A). However, the levels of ERK phosphorylation was further enhanced by M3G treatment compared to UVA-irradiated non-treated HDFs. At 25 μM, ERK activation was 93% higher compared to UVA-irradiated only group.

Parallel to its effects in UVA-irradiated HaCaT keratinocytes, effects of M3G on the levels of c-Fos and c-Jun phosphorylation were investigated using Western blotting. Protein levels of phosphorylated c-Fos and c-Jun in both total cell lysates and nuclear fractions of UVA-irradiated HDFs were significantly increased (Figure 8B). Presence of M3G (25 μM) decreased the phosphorylation of c-Fos and c-Jun both in cellular and nuclear fractions of HDFs suggesting that M3G inhibited MMP production via prevention of AP-1 (c-Fos and c-Jun) activation. At 25 μM treatment, phosphorylation of c-Fos and c-Jun was decreased by 41.3% and 57.8%, respectively. Nuclear fractions of M3G treated (25 μM) HDFs contained 80.3% and 87.8% less phosphorylated c-Fos and c-Jun compared to UVA-irradiated only group.

### 2.10. Effect of M3G on TGFβ/Smad Signaling in UVA-Irradiated HDFs

**Figure 9:**
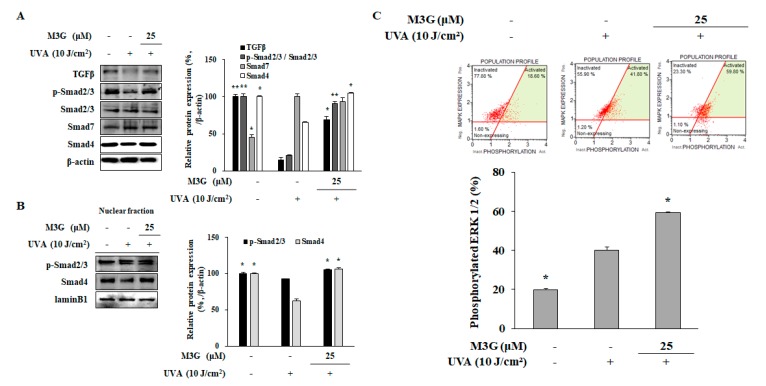
Effect of M3G on the TGFβ/Smad signaling activation in UVA-irradiated HDFs. Cells were exposed to UVA radiation (10 J/cm^2^) and treated with 25 μM of M3G. After 24 h incubation cells were harvested and the protein levels of TGFβ, phosphorylated (p-) and inactive Smad 2/3, Smad 7 and Smad 4 in whole cell lysates (**A**), and p-Smad 2/3 and Smad 4 levels in nuclear fractions (**B**) were determined using Western blotting. β-actin and lamin B1 (nuclear fraction) was used as an internal control for Western blotting. Activation levels of ERK1/2 in UVA-irradiated HDFs treated and non-treated with M3G (25 μM) were analyzed by flow cytometry and given as percentage of cell population with activated ERK1/2 (**C**). * *p* < 0.05, ** *p* < 0.01 compared to UVA-irradiated untreated control.

To investigate the underlying mechanism of M3G-mediated increase in collagen production of UVA-irradiated HDFs, expression and phosphorylation levels of key collagen synthesis cascade (TGFβ, Smad 2/3, Smad 7 and Smad 4) were analyzed using Western blotting. Protein levels of TGFβ and Smad 4 were significantly reduced in HDFs after UVA (10 J/cm^2^) exposure: 85.2% and 34.5%, respectively (Figure 9A). This was also accompanied by diminished phosphorylation of Smad 2/3 complex (79.2%). On the other hand, levels of Smad 7, negative regulator of TGFβ signaling, were observed to be increased by UVA exposure (by 119.8% of non-irradiated non-treated control). M3G treatment (25 μM) reversed the effects of UVA in type I collagen production cascade by increasing the protein levels of TGFβ (368.2%) as well as its downstream proteins Smad 4 (59.2%). In addition, phosphorylation of Smad 2/3 was significantly upregulated (338.55% of UVA-irradiated only group). Detrimental effects of UVA exposure were further showed by the decrease in phosphorylated Smad 2/3 (17.4%) and Smad 4 (37.2%) levels in the nuclear fraction of HDFs (Figure 9B). M3G treatment (25 μM) resulted in the elevation of phosphorylated Smad 2/3 levels by 13.7% and Smad 4 levels by 68.9% compared to UVA-irradiated only group.

To confirm the enhancement of ERK activation by M3G which has been suggested to be related to its ability to stimulate TGFβ activation, ERK1/2 activation was analyzed by flow cytometry. Results showed that M3G treated (25 μM) cells expressed 59.80% activated population in terms of ERK1/2 compared to 41.80% of UVA irradiated only cells (Figure 9C). Results suggested that M3G inhibited the activation of p38 and JNK MAPKs but contradictorily enhanced the ERK activation. Overall, it was demonstrated that M3G relieved the UVA-induced suppression in TGFβ signaling pathway via increasing ERK1/2 phosphorylation which potentially increased collagen synthesis.

## 3. Discussion

UVA rays can penetrate deeper than UVB until the bottom layers of dermis; hence, affect different layers of skin and cell types such as keratinocytes of epidermis and fibroblasts of dermis [2]. Subtoxic UVA exposure had been shown to induce several detrimental effects in both epidermis and dermis resulting in complications such as extrinsic skin aging, also known as photoaging [1]. Photoaging mechanism is complex and not fully understood. However, it had been shown that MMPs had crucial functions in photoaging due to their duties in degrading ECM components. UV irradiation deteriorates the balance between MMP production and ECM formation in favor of MMPs which results in increased degradation of collagen and elastin backbone of ECM. MMP-1 is a collagenase member of MMP family and the first actuator of collagen degradation [4]. Changes in cellular processes induced by UVA upregulate the expression and activation of MMP-1 which triggers the collagen degradation through cleavage of type I collagen. Collagen breakdown is then finalized with the activities of MMP-2 and -9 [4,5,22]. Activation of collagenase MMPs such as MMP-1 and -9 is carried out by the activity of MMP-3 [22]. This cascade of activation and degradation plays the central role in UV-induced photoaging. Many antioxidant flavonoids had been reported to possess MMP inhibitory properties as well as inhibitory effects on MMP production. Myricetin, a flavanol type of flavonoid, had been shown to possess antioxidant capabilities as well as MMP inhibitory effects [23,24]. Thus, present study suggested that M3G, a glycoside derivative of myricetin could prevent UVA-induced overproduction of MMP-1. Retinoic acid has been used as a positive control for the experiments given that it is a well-known antiphotoaging molecule [25]. Results have demonstrated that M3G markedly inhibited the MMP-1 release and expression in UVA-irradiated HaCaT keratinocytes and HDFs. Treatment with M3G not only has downregulated the MMP-1 expression, but also has decreased the protein levels of MMP-9 and MMP-3.

Several studies had confirmed the involvement of the activation of AP-1 transcription factor as a way to express MMPs. UV mediated upregulation of MMP expression had also been reported to be linked with the activation of AP-1, a heterodimer of phosphorylated c-Fos and c-Jun proteins [10]. During the photoaging progression, a sharp increase in ROS and RNS production is observed. Elevated production of reactive species is known to be an activator of MAPK signaling which is also the upstream activator for AP-1 [26]. Hence, UV-induced breakdown of collagen via MMPs is suggested to be regulated by the overactivation of MAPKs. The p38, ERK and JNK MAPKs are three main components of the MAPK cascade and stimulate the AP-1-induced transcriptional activity. Present study has demonstrated that M3G treatment had managed to suppress the activation of these three MAPKs in UVA-irradiated HaCaT keratinocytes. Suppression of p38, ERK and JNK MAPKs by M3G in HaCaT keratinocytes has also been accompanied by the downregulation of UV-induced increase in c-Fos and c-Jun activation, as expected. Therefore, inhibition of UVA-induced MAPK activation has been suggested as the underlying mechanism for the inhibitory effect of M3G on MMP production. In HDFs, however, M3G treatment has enhanced the activation of ERK1/2 despite downregulating AP-1 activation. Overall, results have suggested that M3G inhibited the UVA-induced MMP-1 production in both keratinocytes and dermal fibroblasts via suppression of MAPK-mediated AP-1 activation. ERK1/2 activation had been shown to be a part of collagen synthesis in HDFs [27]. Hence, the opposite effect of M3G on ERK1/2 compared to other MAPKs (p38 and JNK) has been suggested to be part of collagen production stimulatory mechanism.

UV-induced upregulation of MMP production is concomitant to diminished collagen synthesis. Collagens are the main ECM components that are responsible for the strength of the skin. In this context, photoaging is characterized by excessive collagen degradation and decreased collagen synthesis. Results have showed that M3G increased the production of type I procollagen in both HaCaT keratinocytes and HDFs after UVA irradiation. Therefore, current study has suggested a potential antiphotoaging effect for M3G due to attenuation of UVA-induced changes on MMP-1 and collagen production. Production of type I collagen, one of the most common collagen types of dermis layer, is regulated by a downstream signaling of TGFβ/Smad receptors [28]. Activated Smad2/3 complex and Smad4 protein initiate the transcriptional activation of collagen production. M3G treatment of UVA-irradiated HDFs has exerted a relieving effect on the suppression of TGFβ signaling suggested by the increase in phosphorylation of Smad2/3 and the phosphorylated Smad2/3 levels in the nuclear fraction. Therefore, the mechanism behind the upregulation of collagen production by M3G has been suggested to be the activation of TGFβ/Smad pathway. In addition, ERK1/2 MAPK had been suggested to play roles in TGFβ mediated collagen production [27]. The increase in the phosphorylation of ERK1/2 by M3G treatment despite the suppression of p38 and JNK MAPKs has been suggested to be related to its effects on collagen production. It has been hypothesized that M3G upregulated the collagen production via TGFβ-mediated ERK1/2 activation [29].

Inflammation of the skin is another result of UV exposure and contribute significantly to the extrinsic skin aging process. UVA exposure is known to stimulate generation of reaction species. Among them RNS, mainly NO and nitric dioxide are responsible for inflammatory response [30]. Inflammatory response to UVA irradiation involves the production of pro-inflammatory enzymes and cytokines such as COX-2, iNOS, TNF-α, IL-1β and IL-6 [31,32]. Inflammation of skin also increases the ROS production and therefore enhances the UVA-mediated damages in skin cells [4]. Results of the current study have demonstrated that M3G treatment not only decreased the generation of NO but also downregulated the UVA-induced production of COX-2, iNOS, TNF-α, IL-1β and IL-6 in both HaCaT keratinocytes and HDFs. It has been suggested that antiphotoaging effect of M3G against UVA-irradiation in keratinocytes and dermal fibroblasts included the attenuation of inflammatory response. This effect was suggested to be due to its scavenging ability against UVA-induced ROS and RNS which is common for flavonoids.

Flavonoids are known to exert strong antioxidant properties along other bioactivities such as MMP inhibition [12]. Myricetin, flavonoid base of M3G, had been shown to possess antiphotoaging properties by protecting skin from UV-induced damages via its antioxidant [33] and MMP inhibitory capabilities [34]. Studies had reported that these photoprotective effects of myricetin, and other structurally similar flavonoids also suppressed the MAPK activation through ERK [34] and p38 pathway [33]. Current findings have been of similar nature indicating that the reactive species scavenging and MAPK inhibitory effects of M3G resulted in reversing the UVA-induced changes in HaCaT and HDFs. Similar to myricetin, quercetin had also been reported to possess similar properties in addition to its strong anti-inflammatory effects against TNFα mediated inflammatory cytokines [35]. Overall, current results have been in agreement with other flavonoid base antiphotoaging studies and reported the potential in vitro antiphotoaging effect of M3G against UVA irradiation for the first time.

## 4. Materials and Methods

### 4.1. Myricetin 3-O-β-d-Galactopyranoside

Isolation and characterization of myricetin 3-O-β-d-galactopyranoside (M3G) has been previously reported [36]. A stock solution of 1 mM M3G in 10% DMSO (*v*/*v*, in distilled water) was prepared and stored at −20 °C until use.

### 4.2. HaCaT Keratinocyte and Human Dermal Fibroblast (HDF) Culture and Maintenance

HaCaT cells (300493; Cell Line Service, Eppelheim, Germany) were cultured in Dulbecco’s modified Eagle medium with 10% fetal bovine serum (FBS). Cells were kept in 37 °C incubators with an atmosphere containing 5% CO_2_ between the experiments.

HDF cells (C-12302; PromoCell, Heidelberg, Germany) were cultured in Fibroblast Growth Medium (C-23020, PromoCell) and kept in 37 °C incubators with an atmosphere containing 5% CO2 between the experiments.

### 4.3. Cytotoxicity Assay

Any possible toxic effect of M3G and DMSO in keratinocytes and dermal fibroblasts was investigated by colorimetric MTT assay as previously described [16]. Briefly, HaCaT cells and HDFs were seeded in 96-well plates (1 × 10^3^ cell/well) and treated with final concentrations of 1, 5, 10, 20, 25 and 50 μM M3G or equivalent amount of 10% DMSO (*v*/*v*, in distilled water). Culture medium was replaced with 100 μL MTT solution (1 mg/mL) following a 24 h incubation. The plate was then kept in dark at 37 °C for 4 h. Wells were aspired, and cells were washed with phosphate buffer saline (PBS). Ten microliters of 100% DMSO was introduced to each well to dissolve the formazan crystals and absorbance values of wells at 540 nm were measured using GENios FL microplate reader (Tecan Austria GmbH, Grodig, Austria). Same procedure was followed to investigate the effect of UVA exposure on the viability of HaCaT keratinocytes and HDFs. Cells were irradiated by UVA (0–10 J/cm^2^) (Section 4.3) and the cell viability assay was carried out as mentioned without sample treatment. Changes in the viability of cells were calculated as a percentage of the untreated blank group which was considered to be 100% alive and compared to each concentration of M3G treatment.

### 4.4. UVA Irradiation of HaCaT Keratinocytes and HDFs

HaCaT keratinocytes and HDFs were irradiated by UVA using a Bio-Sun UV Irradiation System (Vilber Lourmat, Marine, France) fitted with a UVA source designed for microplates. Cells grown in microplates were irradiated at a sublethal dose of 10 J/cm^2^ UVA. Briefly, cells were irradiated in phosphate-buffered saline (PBS) without the plastic lid using the Bio-Sun system illuminator with a UV peak at 365 nm. Cells were cultured in plates and incubated for 24 h prior to UVA irradiation. Irradiation was emitted from 30W T-20.L tubes (Vilber Lourmat) placed at a distance of 10 from the sample loading tray. Irradiation was carried out automatically by the programmable microprocessor which stopped the irradiation when the energy received matched the desired programmed energy (0–10 J/cm^2^; range of measurement, 0–20 J/cm^2^). Subsequently the cells were incubated with their own growth medium without FBS until analysis.

### 4.5. Enzyme-Linked Immunosorbent Assay (ELISA)

Releases of MMP-1 and type Iα1 procollagen in UVA-irradiated HaCaT keratinocytes and HDFs were analyzed by ELISA. Cells (1 × 10^6^ cell/well) were pre-incubated in 6-well plates for 24 h and washed with PBS prior to UVA (10 J/cm^2^) exposure. Immediately after UVA irradiation, the cells were treated with or without different doses (final concentrations of 1, 5, 25 μM) of M3G and incubated for another 24 h. Cell culture medium from each well was harvested and analyzed for its MMP-1 and type Iα1 procollagen contents per manufacturer’s instructions of the ELISA kit (R&D Systems, Inc., Minneapolis, MN, USA).

### 4.6. Measurement of Nitrate

UVA-induced NO generation by HaCaT keratinocytes and HDFs was quantified by measuring the nitrate amount in conditioned cell culture medium. Cell culture medium was harvested following 24 h incubation after UVA irradiation and sample treatment and centrifuged for 15 min (134× *g*). Supernatants were then filtered through 0.2 μM syringe filter and the nitrate amount was calculated using modified Griess reaction as previously described [37].

### 4.7. Reverse Transcription Polymerase Chain Reaction (RT-PCR) and Quantitative Reverse Transcription Polymerase Chain Reaction (RT-qPCR) Analysis

HaCaT keratinocytes and HDFs cultured in 6-well plates (1 × 10^6^ cell/well) were incubated for 24 h and treated with or without M3G (final concentrations of 1, 5, 25 μM) immediately after UVA (10 J/cm^2^) irradiation, and incubated for another 24 h. Total RNA was isolated from nonirradiated and irradiated (UVA, 10 J/cm^2^) HaCaT keratinocytes and HDFs using TRIzol^®^ reagent (Invitrogen; Thermo Fisher Scientific, Inc., Rockford, IL, USA). Total RNA (2 μg) and oligo(dT) were mixed in RNase-free water for the cDNA synthesis. Synthesis was performed in a thermo cycler (T100; Bio-Rad Laboratories, Inc., Hercules, CA, USA) with an initial denaturation of the mix at 70 °C for 5 min and cooling immediately followed by the preparation of a master mix containing 1× RT buffer, 1 mM dNTPs, 500 ng oligo(dT), 140 units M-MLV reserve transcriptase and 40 units RNase inhibitor. Next, the cDNA synthesis was carried out with two steps of 42 °C for 60 min and 72 °C for 5 min.

For RT-PCR analysis, the target cDNA was amplified using the sense and antisense primers as previously noted [16]. The amplification was carried out with 30 cycles; each cycle consisted of 95 °C for 45 s, 60 °C for 1 min and 72 °C for 45 s. The final PCR products were separated by agarose gel (1.5%) electrophoresis for 30 min at 100 V. Gels were then stained with 1 mg/mL ethidium bromide and visualized by UV light using a CAS-400SM Davinch-Chemi imager™ (Davinch-K, Seoul, Korea).

Gene expression was also measured by RT-qPCR in a Thermal Cycler Dice^®^ Real Time System TP800 (Takara Bio Inc., Ohtsu, Japan) following the manufacturer’s protocol. Briefly 1.0 μL of cDNA sample and 10.5 μL of Luna^®^ Universal qPCR Mix (New England Biolabs, Ipswich, MA, USA) were mixed with forward and reverse primers in nuclease-free water. The target cDNA was amplified using following forward and reverse primers; MMP-1, forward 5‘-TCT GAC GTT GAT CCC AGA GAG CAG-3′, reverse 5‘-CAG GGT GAC ACC AGT GAC TGC AC-3′; type I procollagen, forward 5’-CTC GAG GTG GAC ACC ACC CT-3’, reverse 5’-CAG CTG GAT GGC CAC ATC GG-3’; β-actin, forward 5′-AGA TCA AGA TCA TTG CTC CTC CTG-3′, reverse 5′-CAA GAA AGG GTG TAA CGC AAC TAA G-3′. The PCR amplification was carried out with an initial denaturation at 95 °C for 1 min, followed by 40 PCR cycles, each cycle consisting of 95 °C for 15 s and 60 °C for 30 s. Relative quantification was calculated using the 2^−(ΔΔCT)^ method. β-Actin was used as an internal control.

### 4.8. Western Blotting

Protein levels were investigated using immunoblotting according to common Western blotting protocols. HaCaT keratinocytes and HDFs cultured in 6-well plates (1 × 10^6^ cell/well) were incubated for 24 h and were treated with or without M3G (1, 5, 25 μM) immediately after UVA (10 J/cm^2^) irradiation and incubated for another 24 h. Following incubation, wells were aspirated, and cells were lysed by vigorous pipetting in 1 mL of RIPA buffer (Sigma–Aldrich, St. Louis, MO, USA) at 4 °C to obtain total cell lysates. The nuclear fraction extraction was carried out using NE-PER^TM^ Nuclear Extraction Kit (Catalog No. #78835; Thermo Fisher Scientific) according to manufacturer’s instructions. Protein content of the lysates was measured with a BCA protein assay kit (Thermo Fisher Scientific, Rockford, IL, USA) following kit’s protocol. Same amount (20 μg) of protein from each well was loaded onto 10% SDS-polyacrylamide gel and run at 100 V. Proteins on gel were then transferred onto a polyvinylidene fluoride membrane (Amersham, GE Healthcare, Little Chalfont, UK) using a wet system run at 100 V for 1 h at 4 °C. Membranes were then incubated for 1 h at room temperature in 5% skimmed milk for blocking. Blocked membranes were washed with 1× TBST and incubated with primary antibodies of specific proteins (diluted 1:1000) in primary antibody dilution buffer containing 1× TBST with 5% bovine serum albumin overnight at 4 °C. Membranes were then incubated with horseradish-peroxidase-conjugated secondary antibodies (diluted 1:1000) specific to the primary antibody at room temperature for 1 h. β-actin and lamin B1 were used as internal housekeeping control for total cell lysate and nuclear fractions, respectively. Detection of proteins on blotted membranes was achieved using an ECL Western blot detection kit (Amersham) according to the manufacturer’s instructions. Protein bands were imaged with the CAS-400SM Davinch-Chemi imager™ (Davinch-K, Seoul, Korea).

### 4.9. Flow Cytometry

The MAPK (ERK1/2) activation levels were investigated employing flow cytometry. Cells were pre-incubated in 6-well plates (1 × 10^6^ cell/well) for 24 h and washed with PBS prior to UVA (10 J/cm^2^) exposure. Following UVA irradiation, the cells were treated with or without different concentrations (1, 5, 25 μM) of M3G for 24 h. Levels of ERK1/2 phosphorylation were measured with MUSE^TM^ MAPK Activation Dual Detection Kit (MCH200104; Merck KGaA, Darmstadt, Germany) using MUSE^TM^ Cell Analyzer and software (Muse Cell Soft V1.4.0.0, Merck KGaA, Darmstadt, Germany) according to the manufacturer’s instructions.

### 4.10. Statistical Analysis

All numerical data were given as the mean ± standard deviation of three separate experiments carried out in triplicates. Statistical differences between the means of the sample groups were calculated by the analysis of variance (ANOVA) followed by Duncan’s multiple range test using SAS v9.1 software (SAS Institute, Cary, NC, USA). Any statistically significant difference between the groups was determined at *p* < 0.05 and *p* < 0.01 levels.

## 5. Conclusions

In conclusion, the current study has reported the in vitro anti-photoaging effects of M3G in UVA-irradiated HaCaT keratinocytes and HDFs for the first time. Results have showed that M3G attenuated the UVA-induced overproduction of MMP-1 and suppression of collagen production through inhibition of MAPK signaling and activation of TGFβ signaling, respectively. In addition, M3G has been able to revert UVA-induced inflammation in vitro by suppressing pro-inflammatory cytokines. Therefore, M3G has been suggested to be a promising flavonol derivative with potential antiphotoaging properties. Further studies for the utilization of M3G in cosmeceutical applications against UVA irradiation were also suggested to be carried out in animal models to elucidate its action mechanisms and additional beneficial effects.
